# An Investigation of 2′-Deoxyribonucleoside Cyanoboranes in Mice for Therapeutic Safety

**DOI:** 10.1155/MBD.1994.19

**Published:** 1994

**Authors:** Iris H. Hall, Bruce S. Burnham, K. G. Rajendran, J.-J. Chang, Anup Sood, Bernard Spielvogel

**Affiliations:** 1 Division of Medicinal Chemistry and Natural Products School of Pharmacy, CB# 7360 University of North Carolina Chapel Hill Chapel Hill NC 27599 USA; 2 Department of Pathology School of Medicine University of North Carolina Chapel Hill Chapel Hill NC 27599 USA; 3 Boron Biologicals, Inc. 533 Pylon Drive Raleigh NC 27636-3489 USA

## Abstract

A standard acute toxicity study was undertaken to assess 2′-deoxyribonucleoside cyanoboranes for therapeutic safety. 2′-Deoxyribonucleoside cyanoboranes and related derivatives were nontoxic at doses required for anti-neoplastic and hypolipidemic activities. At higher doses (50 and 100 mg/kg/day IP for 7 days), all treated animals survived with slight reductions in total body weight and small decrements in daily food consumption. No clinical chemistry value was elevated to a magnitude suggesting onset of organ specific toxicity. However, agents appeared to modulate subpopulations of white blood cells, i.e., more lymphocytes than PMNs were present in blood from treated animals as determined by differential cell counts. This modulation is correlated with increases in granulomatous foci in the spleen and mesentery of treated animals after 7 days. The kidney was damaged only by Compound 5 at 50 and 100 mg/kg/day; Compound 5 had the most potent anti-neoplastic activity. The compounds demonstrated no in vitro toxicity against human HCT-8 ileum cells. LD_50_ values were greater than 1000 mg/kg, IP, for all compounds.


### AN INVESTIGATION OF 2'-DEOXYRIBONUCLEOSIDE CYANOBORANES IN MICE FOR THERAPEUTIC SAFETY

Iris H. Hall[1], Bruce S. Burnham[1], K.G. Rajendran[1], J.-J. Chang[2], Anup Sood[3] and Bernard Spielvogel[3]

[1] Division of Medicinal Chemistry and Natural Products, School of Pharmacy, CB# 7360 University of North Carolina - Chapel Hill, Chapel Hill, NC 27599, USA
[2] Department of Pathology, School of Medicine, University of North Carolina - Chapel Hill Chapel Hill, NC 27599, USA
[3] Boron Biologicals, Inc., 533 Pylon Drive, Raleigh, NC 27636-3489, USA

**Abstract**

A standard acute toxicity study was undertaken to assess 2'-deoxyribonucleoside cyanoboranes for therapeutic safety. 2'-Deoxyribonucleoside cyanoboranes and related derivatives were nontoxic at doses required for anti-neoplastic and hypolipidemic activities. At higher doses (50 and 100 mg/kg/day IP for 7 days), all treated animals survived with slight reductions in total body weight and small decrements in daily food consumption. No clinical chemistry value was elevated to a magnitude suggesting onset of organ specific toxicity. However, agents appeared to modulate subpopulations of white blood cells, i.e., more lymphocytes than PMNs were present in blood from treated animals as determined by differential cell counts. This modulation is correlated with increases in granulomatous foci in the spleen and mesentery of treated animals after 7 days. The kidney was damaged only by Compound 5 at 50 and 100 mg/kg/day; Compound 5 had the most potent anti-neoplastic activity. The compounds demonstrated no in vitro toxicity against human HCT-8 ileum cells. $LD_{50}$ values were greater than 1000 mg/kg, IP, for all compounds.

**Introduction**

2'-Deoxyribonucleoside cyanoboranes have been investigated for cytotoxic activity against murine and human tumor cell growth, and for their anti-neoplastic activity against murine tumors [1,2]. They have also recently been observed to be hypolipidemic agents, significantly inhibiting enzymes involved in de novo lipid synthesis [3]. Moreover, these same derivatives were recently shown to protect against LPS-induced septic shock. The guanine and cytidine derivatives have proven to be pharmacologically more active than some of the other bases, e.g. inosine, adenine, or thymidine [1]. The 8 and 20 mg/kg/day doses used in these pharmacological screens demonstrated no deleterious effects in intact animals [1-3]. Thus we have selected 20 mg/kg as the standard therapeutic dose. Because of their possible therapeutic value, five 2'-deoxyribonucleoside cyanoboranes were selected to assess acute toxicity in mice in vivo. Deleterious effects were evaluated at dosage levels of 20, 50, and 100 mg/kg and also in HCT-8 human ileum mucosa cells grown in tissue culture.

**Materials & Methods**

Compounds 1-5 (Fig. 1), have been synthesized previously; chemical and physical characteristics have been reported [1,2]. Suspensions of compounds were prepared by homogenizing them in 0.05% Tween 80/$H_2O$. Controls received injections of vehicle throughout the 7-day experiment.

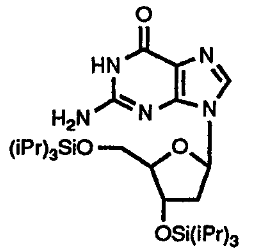

1

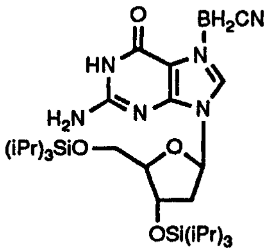

2

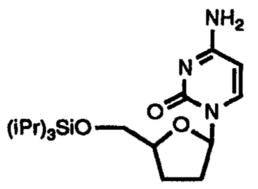

3

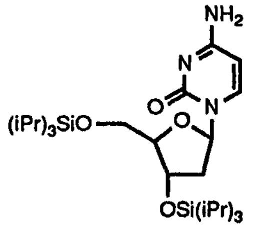

4

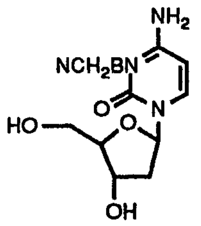

5

Figure 1. Structures of 2'-Deoxyribonucleoside and Cyanoborane Derivatives.

$CF_1$ male mice (~28 g, *N*=5) were dosed with Compounds 1-5 at 20, 50, and 100 mg/kg/day IP for 7 days [4]. Blood was obtained from the carotid artery and centrifuged at 3500 g x 10 min to obtain serum. Daily food consumption was determined; water was available *ad libitum*. Animals were maintained in 12 h light:dark cycles at $72^{o}F$.

At the time of sacrifice, the final body weight was obtained and the major organs were excised, trimmed of fat and weighed. Chemical or enzymatic assays were performed with Sigma Chemical kits (St. Louis, MO, USA): urea nitrogen (BUN, No. 640), alanine amino transferase (SGPT, No. 505), glucose (No. 510), creatine phosphokinase (CP-kinase, No. 661), lactic dehydrogenase (LDH, No. 500), total and direct bilirubin (No. 605), aldolase (No. 752), calcium (No. 587 and Arsen A20 111), and alkaline phosphatase (AP, No. 104). Serum cholesterol was determined by the method of Ness et al. [5]. Serum triglycerides were determined with a diagnostic kit from Boehringer Mannheim (Diagnostics, Indianapolis, IN, USA). Uric acid and cholic acid were determined as outlined by Tietz [6]. Albumin and total protein were determined by the method of Lowry, et al. [7].

After blood was obtained from the carotid artery of each $CF_1$ mouse, it was placed on a glass slide and fixed in Wright's stain. Differential white blood cell counts, platelet counts and hematocrits were obtained after sacrificing each group at the specified times [8].

After adminstration of each dose of the 5 compounds or their respective controls, $CF_1$ mice were killed by carbon dioxide asphyxiation. After all vital signs had ceased, a midline incision was made from the lower jaw to the inguinal area. The spleen, liver, and kidney were excised and weighed, and then representative tissue samples (6 sections randomly selected from each group) were fixed in 10% buffered formalin, trimmed and sectioned at 6µ in thickness and stained with hematoxylin and eosin. Abnormal histological/morphological findings were ascertained with light microscopy.

To evaluate in vitro toxicity, HCT-8 human ileum cells (American Tissue Culture Cells) were maintained in RPMI-1640 supplemented with L-glutamine, 10% fetal calf serum (FCS) and penicillin/streptomycin (P/S) and incubated for 18 hr with agents at $10^{-4}$ M. Cell growth was determined by 2% crystal violet in 20% MeOH [9]. Growth in the wells was determined at 580 nm in a Molecular Devices scanner (SOFTmax Program). The medium was harvested and the following enzyme activities were determined: lactic dehydrogenase [10], AP [11,12], trypsin [13], leucine amino peptidase [14], α-galactosidase [9,15], and cAMP ($^{125}$I-RIA-New England Nuclear kit). Protein levels were determined by the Lowry et al. method [7].

To evaluate in vivo toxicity, compounds 1-5 were administered to $CF_1$ male mice as a single injection IP at 100, 250, 500, 1000, 1500, and 2000 mg/kg. Six mice received each dose. Mice were weighed daily and the number of deaths was recorded each day during a 14-day period [16]. $LD_{50}$ values were determined by the Probit procedure [17].

Student's "t" tests were conducted between control and treatment groups. Analyses of variance (ANOV), followed by Duncan's multiple range tests, were conducted for comparisons between treatment groups.

**Results**

After administration of *Compound 1* at 20, 50, or 100 mg/kg/day IP for 7 days, all animals survived without decreases in daily food consumption. Total body weight decreased as the dose increased with an approximate 15% reduction from the control values at the highest dose. The hematocrits (%) were unchanged for all doses (Table I).

Weights of the brain, lung, and thymus decreased with increasing drug doses. Weights of the liver, spleen, heart, kidney, and small intestine did not differ from control weights at any dose employed. Weights of the stomach, large intestine, and reproductive organs varied with drug dose but with no obvious correlation to the dose used. Histological examinations revealed that all kidney sections from animals treated at 20 mg/kg/day showed multiple foci of hemorrhage in the cortex with cellular loss. No necrotic nuclei in these sections were found. Interestingly, at the 50 and 100 mg/kg/day doses, the kidney sections appeared normal. Spleen sections (66%) at the 20 mg/kg/day dose showed several small fibrous plaques on the serosal surface. One-third of the spleens at the 50 mg/kg/day dose contained fibrous plaques; and at 100 mg/kg/day, all spleen sections contained chronic granulomatous inflammatory foci or surface fibrosal plaques. Hepatic histology from mice administered the 20 mg/kg/day dose revealed that one-third of the sections had collections of acute and chronic inflammatory cells at the portal triad. At the 50 mg/kg/day dose, one-third of the livers contained at least one fibrous plaque on the serosal surface; and at the 100 mg/kg/day dose, all liver sections had one minor granulomatous focus.

Clinical chemistry values demonstrated some variability among treatment groups; however, none of the changes suggested any organ specific toxicity (Table I). Compound 1 did cause a significant reduction in BUN and bilirubin levels. Elevations were observed in serum calcium levels. CP kinase was elevated at 20 mg/kg/day, but actually decreased markedly at 50 and 100 mg/kg/day. For the differential white blood cell count, there was a drug-induced increase in lymphocytes and a decrease in PMNs.

Treatment with *Compound 2* resulted in all animals surviving at all three doses (Table II). Only the 100 mg/kg/day dose reduced daily food consumption. Treated groups showed decreases in total body weight rather than the weight increases usually seen, but the effect was not dose-dependent. Hematocrit (%) values did not vary in the treatment groups.

Some variability occurred in individual organ weights with some exhibiting marked alterations; however average heart, brain, lung, stomach, and reproductive organ weights were relatively stable. Weights of the liver and thymus increased after treatment with 100 mg/kg/day. Small intestine weights were increased at all three doses; conversely, large intestine weights were decreased at all three doses. Histologically at the 20 mg/kg/day dose,

**Table I. Acute Toxicity of 1 3',5'-o-bis(triisopropylsilyl)-2'-deoxyguanosine**

| *COMPOUND 1* | Control | 20 mg/kg | 50 mg/kg | 100 mg/kg |
|---|---|---|---|---|
| **General** | | | | |
| Wt. Gain over 7 Days | 109.0±1.50 | 97.70±1.20 | 101.6±1.30 | 94.6±1.4 |
| Food Consumption | 18.4±0.90 | 18.10±1.10 | 20.3±0.85 | 19.2±1.2 |
| Survival | 5 of 5 | 5 of 5 | 5 of 5 | 5 of 5 |
| Hematocrit | 50.8±0.80 | 46.4±0.90 | 48.0±0.70 | 47.2±0.5 |
| **Organ Wts. (g/100 g body wt)** | | | | |
| Brain | 1.675±0.089 | 1.473±0.111 | 1.437±0.104 | 1.381±0.098 |
| Heart | 0.511±0.041 | 0.526±0.054 | 0.548±0.036 | 0.569±0.039 |
| Lung | 0.819±0.052 | 0.751±0.058 | 0.739±0.052 | 0.612±0.064 |
| Thymus | 0.400±0.040 | 0.377±0.032 | 0.364±0.038 | 0.248±0.035* |
| Liver | 4.570±0.076 | 5.200±0.064 | 5.245±0.062 | 5.428±0.079 |
| Spleen | 0.318±0.070 | 0.496±0.062 | 0.455±0.079 | 0.656±0.083 |
| Kidney | 1.330±0.048 | 1.378±0.049 | 1.383±0.057 | 1.328±0.041 |
| Stomach | 2.096±0.053 | 1.529±0.062* | 1.928±0.068 | 2.699±0.059* |
| Small Intestine | 5.992±0.089 | 5.011±0.092 | 5.865±0.086 | 5.934±0.081 |
| Large Intestine | 4.043±0.089 | 3.942±0.076 | 3.824±0.072 | 4.427±0.064 |
| Reproductive Organs | 1.481±0.062 | 1.871±0.052 | 2.093±0.051* | 2.257±0.068* |
| **Clinical Chemistry** | | | | |
| BUN (mg/dL) | 100±5 | 79±4* | 98±5 | 79±6* |
| SGPT (mmol/min/L) | 100±7 | 100±7 | 100±6 | 99±5 |
| Glucose (mg/dL) | 100±5 | 101±5 | 86±7 | 102±6 |
| CP kinase (Sigma unit/L) | 100±5 | 145±5* | 60±5* | 61±4* |
| LDH (Sigma unit/L) | 100±6 | 59±6* | 68±4* | 121±3 |
| Bilirubin (g/100 mL) | 100±4 | 67±4* | 91±6 | 88±4 |
| Cholesterol (mg/dL) | 100±5 | 68±6* | 106±6 | 104±5 |
| Triglyceride (mg/dL) | 100±6 | 93±3 | 101±5 | 110±6 |
| Aldolase | 100±6 | 87±5 | 92±6 | 91±6 |
| Uric Acid (g/100 mL) | 100±7 | 103±4 | 122±7 | 95±5 |
| Calcium (mg/dL) | 100±5 | 137±7 | 132±6 | 123±4 |
| Alkaline Phosphatase (μmol/min/L) | 100±3 | 96±6 | 102±5 | 102±3 |
| Cholic Acid (mg/dL) | 100±4 | 98±5 | 126±11 | 113±4 |
| Total Protein (g/100 mL) | 100±5 | 94±5 | 101±4 | 83±5 |
| **Differential White Blood Cells (%)** | | | | |
| Lymphocytes | 57%±5 | | 65%±5 | 73%±5 |
| PMNs | 42%±5 | | 23%±3 | 25%±4 |
| Basophils | 1%±1 | | 0%±0 | 1%±0 |
| Eosinophils | 0%±0 | | 0%±0 | 0%±0 |
| Monocytes | 0%±0 | | 1%±0 | 1%±0 |

$P \leq 0.001$

33% of the mesenteric mesenchymal and fatty tissues contained exudates consisting of macrophages and lymphocytes with a few PMNs. At the 50 mg/kg/day dose, one hepatic section contained a few granulomatous foci. All other sections were normal, particularly at the 100 mg/kg/day dose of Compound 2.

The lack of changes in clinical chemistry values suggests that Compound 2 caused no organ toxicity (Table II). Bilirubin, cholesterol, and adolase levels were reduced in a dose-

**Table II. Acute Toxicity of 2, 3',5'-o-bis(triisopropylsilyl)-2'-deoxyguanosine-N7-cyanoborane**

| *COMPOUND 2* | Control | 20 mg/kg/day | 50 mg/kg/day | 100 mg/kg/day |
|---|---|---|---|---|
| **General** | | | | |
| Wt. Gain over 7 Days | 101.6±1.70 | 101.6±1.50 | 100.0±1.10 | 101.7±1.40 |
| Food Consumption | 12.6±0.90 | 12.4±0.85 | 12.4±0.80 | 9.4±0.60* |
| Survival | 5 of 5 | 5 of 5 | 5 of 5 | 5 of 5 |
| Hematocrit | 50.3±1.2 | 45.8±1.1 | 46.9±1.3 | 45.3±1.4 |
| **Organ Wts. (g/100 g body weight)** | | | | |
| Brain | 1.620± 0.090 | 1.580± 0.087 | 1.540± 0.088 | 1.520± 0.082 |
| Heart | 0.510± 0.042 | 0.520± 0.045 | 0.490± 0.043 | 0.520± 0.041 |
| Lung | 0.730± 0.055 | 0.890± 0.051 | 0.710± 0.047 | 0.820± 0.057 |
| Thymus | 0.270± 0.038 | 0.240± 0.042 | 0.240± 0.031 | 0.370± 0.027 |
| Liver | 5.480± 0.076 | 5.950± 0.081 | 5.920± 0.071 | 7.260± 0.070 |
| Spleen | 0.390± 0.062 | 0.390± 0.059 | 0.390± 0.051 | 0.520± 0.061 |
| Kidney | 1.440± 0.045 | 1.460± 0.041 | 1.430± 0.042 | 1.600± 0.051 |
| Stomach | 2.140± 0.051 | 2.810± 0.059 | 2.600± 0.053 | 2.330± 0.061 |
| Small Intestine | 5.690± 0.087 | 6.230± 0.092 | 6.210± 0.098 | 6.620± 0.095 |
| Large Intestine | 5.640± 0.065 | 3.790± 0.061 | 3.850± 0.042 | 3.710± 0.053 |
| Reproductive Organs | 0.970± 0.051 | 1.460± 0.042* | 1.110± 0.061 | 1.020± 0.071 |
| **Clinical Chemistry** | | | | |
| BUN (mg/dL) | 100±5 | 106±6 | 102±7 | 118.0±6 |
| SGPT (mmol/min/L) | 100±7 | 89±5 | 89±6 | 90.0±7 |
| Glucose (mg/dL) | 100±5 | 89±6 | 103±5 | 103.2±6 |
| CP kinase (Sigma unit/L) | 100±5 | 69±5* | 73±7* | 104.7±5 |
| LDH (Sigma unit/L) | 100±6 | 55±4* | 62±5* | 115.3±4 |
| Bilirubin (g/100 mL) | 100±4 | 89±6 | 89±6 | 78.1±6 |
| Cholesterol (mg/dL) | 100±5 | 98±6 | 75±6* | 65.0±5* |
| Triglyceride (mg/dL) | 100±6 | 95±5 | 86±5 | 107.0±6 |
| Aldolase | 100±6 | 94±5 | 92±4 | 76.2±4* |
| Uric Acid (g/100 mL) | 100±7 | 109±7 | 114±3 | 110.9±6 |
| Calcium (mg/dL) | 100±5 | 179±6* | 149±6* | 107.5±7 |
| Alkaline Phosphatase (μmol/min/L) | 100±3 | 94±5 | 127±3* | 148.1±7 |
| Cholic Acid (mg/dL) | 100±4 | 106±4 | 104±5 | 104.0±6 |
| Total Protein (g/100 mL) | 100±5 | 94±5 | 92±6 | 95.1±3 |
| **Differential White Blood Cell Count (%)** | | | | |
| Lymphocytes | 56%±4 | | 75%±0 | 82%±0 |
| PMNs | 38%±4 | | 20%±0 | 14%±0 |
| Basophils | 2%±1 | | 3%±0 | 2%±0 |
| Eosinophils | 1%±0 | | 1%±0 | 1%±0 |
| Monocytes | 3%±0 | | 1%±0 | 1%±0 |

* $P \leq 0.001$

dependent manner. CP kinase activity and LDH levels were reduced after treatment at 20 and 50 mg/kg/day; however, these levels remained at control values in animals treated at 100 mg/kg/day. Calcium levels were elevated at 20 and 50 mg/kg/day, but remained at control values at the 100 mg/kg/day dose. AP levels were elevated at the 50 and 100 mg/kg/day doses. Differential white blood cell counts showed an increase in lymphocytes and a decrease in PMNs. The small percentage changes in the other minor white blood cells were not significant. Platelet estimates were normal for all doses.

**Table III: Acute Toxicity of 3, 5'-Triisopropylsilyl-2',3'-dideoxycytitidine**

| *COMPOUND 3* | Control | 20 mg/kg/day | 50 mg/kg/day | 100 mg/kg/day |
|---|---|---|---|---|
| **General** | | | | |
| Wt. Gain over 7 Days | 101.1±1.40 | 101.4±1.20 | 102.5±1.00 | 96.7±0.90 |
| Food Consumption | 13.9±0.80 | 12.5±0.90 | 13.3±0.90 | 14.7±0.90 |
| Survival | 5 of 5 | 5 of 5 | 5 of 5 | 5 of 5 |
| Hematocrit | 49.7±0.90 | 45.0±0.80 | 46.7±0.40 | 45.4±0.80 |
| **Organ Wts. (g/100 g body weight)** | | | | |
| Brain | 1.870±0.088 | 1.050±0.091 | 1.401±0.085 | 1.413±0.082 |
| Heart | 0.493±0.047 | 0.506±0.042 | 0.616±0.049 | 0.532±0.039 |
| Lung | 0.692±0.051 | 0.749±0.045 | 0.692±0.041 | 0.773±0.053 |
| Thymus | 0.281±0.039 | 0.255±0.036 | 0.292±0.025 | 0.232±0.040 |
| Liver | 5.166±0.077 | 5.435±0.071 | 5.895±0.069 | 5.579±0.076 |
| Spleen | 0.426±0.068 | 0.435±0.062 | 0.521±0.053 | 0.484±0.049 |
| Kidney | 1.494±0.042 | 1.362±0.039 | 1.254±0.027 | 1.474±0.031 |
| Stomach | 2.421±0.050 | 2.559±0.032 | 3.263±0.029 | 2.535±0.036 |
| Small Intestine | 6.323±0.091 | 6.602±0.082 | 6.904±0.041 | 6.135±0.086 |
| Large Intestine | 4.844±0.062 | 4.749±0.061 | 3.600±0.059 | 4.524±0.068 |
| Reproductive Organs | 0.828±0.047 | 1.066±0.059 | 1.053±0.042 | 1.772±0.082* |
| **Clinical Chemistry** | | | | |
| BUN (mg/dL) | 100±5 | 80±6* | 87±5 | 96±3 |
| SGPT (mmol/min/L) | 100±7 | 100±6 | 107±7 | 93±7 |
| Glucose (mg/dL) | 100±5 | 97±5 | 96±6 | 88±8 |
| CP kinase (Sigma unit/L) | 100±5 | 98±5 | 113±6 | 68±7* |
| LDH (Sigma unit/L) | 100±6 | 112±7 | 123±5 | 154±7* |
| Bilirubin (g/100 mL) | 100±4 | 100±6 | 118±5 | 119±6 |
| Cholesterol (mg/dL) | 100±5 | 97±5 | 86±4 | 76±4* |
| Triglyceride (mg/dL) | 100±6 | 109±6 | 121±5 | 122±6 |
| Aldolase | 100±6 | 97±4 | 79±4 | 53±5* |
| Uric Acid (g/100 mL) | 100±7 | 111±5 | 120±6 | 110±7 |
| Calcium (mg/dL) | 100±6 | 100±4 | 96±6 | 148±6* |
| Alkaline Phosphatase (µmol/min/L) | 100±3 | 124±4 | 115±55 | 112±7 |
| Cholic Acid (mg/dL) | 100±4 | 139±5* | 151±7* | 111±7 |
| Total Protein (g/100 mL) | 100±5 | 97±6 | 98±6 | 93±6 |
| **Differential White Blood Cell Count (%)** | | | | |
| Lymphocytes | 57%±4 | | 60%±6 | 69%±6 |
| PMNs | 42%±3 | | 38%±3 | 25%±0 |
| Basophils | 1%±0 | | 1%±0 | 2%±0 |
| Eosinophils | 0%±0 | | 0%±0 | 2%±0 |
| Monocytes | 0%±0 | | 1%±0 | 2%±0 |

* $P \leq 0.001$

Survival after treatment with *Compound 3* at three dosages (20, 50, and 100 mg/kg/day) did not differ from that of controls (Table III). There was no change in daily food consumption; however, a 15% decrease in total body weight was evident at the 100 mg/kg/day dose. The hematocrit values (%) were normal at all dosage levels.

**Table IV. Acute Toxicity of 4, 3',5'-o-bis(triisopropylsilyl)-2'-deoxycytidine**

| *COMPOUND 4* | Control | 20 mg/kg/day | 50 mg/kg/day | 100 mg/kg/day |
|---|---|---|---|---|
| **General** | | | | |
| Wt. Gain over 7 Days | 109.4±1.30 | 110.9±1.20 | 114.2±1.40 | 113.4±1.20 |
| Food Consumption | 11.5±0.9 | 10.3±0.8 | 12.5±0.9 | 11.8±1.0 |
| Survival | 5 of 5 | 5 of 5 | 5 of 5 | 5 of 5 |
| Hematocrit | 46.7±1.00 | 44.8±0.80 | 45.0±0.90 | 44.6±0.90 |
| **Organ Wts. (g/100 g body weight)** | | | | |
| Brain | 1.318±0.072 | 1.213±0.069 | 1.266±0.062 | 1.054±0.067 |
| Heart | 0.614±0.041 | 0.667±0.039 | 0.625±0.025 | 0.553±0.027 |
| Lung | 0.787±0.06 | 0.763±0.058 | 0.714±0.051 | 0.656±0.057 |
| Thymus | 0.269±0.027 | 0.304±0.022 | 0.209±0.019 | 0.279±0.033 |
| Liver | 5.986±0.056 | 6.196±0.059 | 6.207±0.063 | 6.693±0.034 |
| Spleen | 0.402±0.039 | 0.672±0.027 | 0.477±0.036 | 0.493±0.047 |
| Kidney | 1.937±0.047 | 1.875±0.039 | 1.969±0.051 | 1.809±0.043 |
| Stomach | 2.136±0.055 | 2.365±0.042 | 2.727±0.049 | 3.051±0.061 |
| Small Intestine | 7.169±0.088 | 7.344±0.08 | 5.453±0.092 | 5.811±0.07 |
| Large Intestine | 4.313±0.072 | 3.269±0.069 | 4.401±0.082 | 3.393±0.065 |
| Reproductive Organs | 2.518±0.048 | 2.876±0.052 | 2.807±0.041 | 2.911±0.045 |
| **Clinical Chemistry** | | | | |
| BUN (mg/dL) | 100±5 | 96±5 | 85±4 | 70±5* |
| SGPT (mmol/min/L) | 100±7 | 104±6 | 97±6 | 95±7 |
| Glucose (mg/dL) | 100±5 | 104±6 | 97±5 | 95±6 |
| CP kinase (Sigma unit/L) | 100±5 | 108±6 | 66±5* | 108±7 |
| LDH (Sigma unit/L) | 100±6 | 154±7 | 116±6 | 113±6 |
| Bilirubin (g/100 mL) | 100±4 | 96±3 | 72±5* | 60±5* |
| Cholesterol (mg/dL) | 100±5 | 95±5 | 107±6 | 119±6 |
| Triglyceride (mg/dL) | 100±6 | 91±6 | 89±7 | 91±7 |
| Aldolase | 100±6 | 95±6 | 91±6 | 84±7 |
| Uric Acid (g/100 mL) | 100±7 | 95±6 | 86±5 | 75±6 |
| Calcium (mg/dL) | 100±5 | 117±7 | 106±5 | 96±5 |
| Alkaline Phosphatase (μmol/min/L) | 100±3 | 80±5 | 90±6 | 94±5 |
| Cholic Acid (mg/dL) | 100±4 | 84±6 | 72±5 | 53±4* |
| Total Protein (g/100 mL) | 100±5 | 99±7 | 104±6 | 99±6 |
| **Differential White Blood Cell Count (%)** | | | | |
| Lymphocytes | 52%±4 | | 59%±4 | 71%±4 |
| PMNs | 45%±3 | | 39%±3 | 26%±3 |
| Basophils | 2%±1 | | 1%±0 | 1%±0 |
| Eosinophils | 0%±0 | | 0%±0 | 0%±0 |
| Monocytes | 1%±0 | | 1%±0 | 1%±0 |

* $P \leq 0.001$

Individual organ weights varied slightly after treatment with 3. Weights of the liver, stomach, thymus, heart, spleen, and small intestine did not differ from control values. Brain weight at the 20 mg/kg/day was reduced; however, it approached control values at the 50 and 100 mg/kg/day doses. Weights of the lungs and reproductive organs increased as the dose increased. The weight of the large intestine decreased at the 50 mg/kg/day dose; it was consistent with control values at the 20 and 100 mg/kg/day doses.

Histology of liver and kidney sections after treatment with Compound 3 at 20, 50 and 100 mg/kg/day doses was normal. However, 67% of spleen and mesenteric tissues showed fibrotic plaques and granulomatous foci. At the 100 mg/kg/day dose, all splenic sections with mesenteric connective tissues contained granulomatous inflammation, although the spleen weight was normal for all doses.

Clinical chemistry values did not reveal any specific drug-related organ toxicity after treatment with Compound 3 (Table III). BUN, cholesterol, aldolase, and CP kinase were reduced in a dose-dependent manner. LDH and triglyceride levels were increased in a similar fashion. AP and cholic acid levels were raised at lower doses but returned to control values at the 100 mg/kg/day dose. In the differential white blood cell count, lymphocytes increased while PMN levels decreased. Platelet estimates were normal at all doses.

*Compound 4* caused no changes in survival of the animals or in total body weight increases measured after treatment for 7 days (Table IV). Although daily food consumption was reduced slightly in mice at the 20 mg/kg/day dose, this parameter was within normal limits at the 50 and 100 mg/kg/day doses. Similarly, hematocrit percentages did not differ from control values.

Individual organ weights were normal for the thymus and kidney at all doses of 4. Weights of the heart, lung, brain, small intestine, and large intestine decreased from control values after treatment at the 100 mg/kg/day dose. Spleen weight increased after treatment at 20 mg/kg/day, but returned to control values at 50 and 100 mg/kg/day doses. Increases in weights of the stomach and reproductive organs were directly related to dosage level, indicating a dose-response relationship. Histological examination revealed no visible morphological lesions in the liver and kidney except in mice receiving the 50 mg/kg/day dose. One kidney section showed minor granulomatous inflammation on the capsular surface. Spleen sections in greater than 66% of the mice from all treatment groups including the control showed minute foci of granulomatous inflammation on the capsular surface.

Clinical chemistry values were altered after treatment with Compound 4 (Table IV). BUN, CP-kinase, bilirubin, uric acid, alkaline phosphatase, and cholic acid showed decreases in values at 100 mg/kg/day. LDH values were elevated at 20 mg/kg/day, and slightly elevated at 50 and 100 mg/kg/day. Serum cholesterol levels were elevated at 100 mg/kg/day, and calcium levels were elevated at 20 mg/kg/day. In the differential white blood cell counts at higher drug concentrations, lymphocytes were increased and PMNs were reduced. Platelet estimates were normal for all groups.

After treatment with *Compound 5*, all of the animals survived at all three doses employed (Table V). The total body weight increase over 7 days was reduced after treatment with the three doses. Yet, daily food consumption was increased after treatment with 20 and 50 mg/kg/day doses. The hematocrit (%) were normal for animals treated with Compound 5 at all dosage levels.

Individual organ weights were altered by Compound 5, e.g. heart, lung, and stomach

**Table V. Acute Toxicity of 5, 2'-deoxycytidine-N3-cyanoborane**

| *COMPOUND 5* | Control | 20 mg/kg/day | 50 mg/kg/day | 100 mg/kg/day |
|---|---|---|---|---|
| **General** | | | | |
| Wt. Gain over 7 Days | 113.8±1.30 | 110.1±1.20 | 104.6±1.40 | 106.7±1.30 |
| Food Consumption | 18.9±0.80 | 22.9±1.20 | 22.9±1.10 | 18.9±1.00 |
| Survival | 5 of 5 | 5 of 5 | 5 of 5 | 5 of 5 |
| Hematocrit | 45.680±0.80 | 43.906±0.60 | 45.519±0.70 | 44.876±0.80 |
| **Organ Wts. (g/100 g body weight)** | | | | |
| Brain | 1.356±0.088 | 1.489±0.071 | 1.335±0.069 | 1.222±0.032 |
| Heart | 0.555±0.043 | 0.476±0.048 | 0.372±0.051 | 0.576±0.050 |
| Lung | 0.875±0.056 | 0.759±0.052 | 0.810±0.048 | 0.829±0.053 |
| Thymus | 0.229±0.027 | 0.287±0.025 | 0.240±0.021 | 0.286±0.029 |
| Liver | 5.211±0.065 | 5.930±0.059 | 5.888±0.061 | 6.046±0.062 |
| Spleen | 0.426±0.062 | 0.621±0.055 | 0.577±0.069 | 0.590±0.053 |
| Kidney | 1.788±0.047 | 1.866±0.041 | 1.906±0.051 | 2.011±0.049 |
| Stomach | 1.907±0.048 | 1.790±0.056 | 1.919±0.038 | 2.142±0.053 |
| Small Intestine | 6.140±0.077 | 4.283±0.071 | 5.907±0.065 | 4.608±0.069 |
| Large Intestine | 4.539±0.057 | 4.864±0.047 | 4.400±0.071 | 4.483±0.067 |
| Reproductive Organs | 2.354±0.062 | 2.321±0.051 | 2.847±0.059 | 2.484±0.077 |
| **Clinical Chemistry** | | | | |
| BUN (mg/dL) | 100±5 | 63±4* | 86±3 | 92±6 |
| SGPT (mmol/min/L) | 100±7 | 100±6 | 99±7 | 101±5 |
| Glucose (mg/dL) | 100±5 | 98±5 | 99±4 | 88±5 |
| CP kinase (Sigma unit/L) | 100±5 | 45±5* | 84±5 | 37±4 |
| LDH (Sigma unit/L) | 100±6 | 84±7 | 88±6 | 86±5 |
| Bilirubin (g/100 mL) | 100±4 | 94±6 | 70±6 | 96±6 |
| Cholesterol (mg/dL) | 100±5 | 92±5 | 86±5 | 101±7 |
| Triglyceride (mg/dL) | 100±6 | 98±7 | 77±6 | 88±6 |
| Aldolase | 100±6 | 42±5* | 55±7* | 43±4 |
| Uric Acid (g/100 mL) | 100±7 | 73±6* | 75±5 | 115±5 |
| Calcium (mg/dL) | 100±5 | 97±7 | 105±6 | 106±4 |
| Alkaline Phosphatase (μmol/min/L) | 100±3 | 122±6 | 148±5* | 136±6 |
| Cholic Acid (mg/dL) | 100±4 | 86±5 | 99±6 | 94±5 |
| Total Protein (g/100 mL) | 100±5 | 95±4 | 108±7 | 107±6 |
| **Differential White Blood Cell Count (%)** | | | | |
| Lymphocytes | 56%±4 | 58%±4 | 64%±4 | 56%±5 |
| PMNs | 38%±4 | 40%±4 | 34%±2 | 41%±4 |
| Basophils | 2%±0 | 1%±0 | 1%±0 | 2%±0 |
| Eosinophils | 1%±0 | 0%±0 | 0%±0 | 1%±0 |
| Monocytes | 3%±1 | 1%±0 | 1%±0 | 0%±0 |
| Rouleux formation | No | No | No | Yes |

* $P \leq 0.001$

weights were reduced at 20 mg/kg/day; these weights returned to normal values at the 50 and 100 mg/kg/day doses. The brain and small intestine were reduced in weight after treatment with the 100 mg/kg/day dose. Weights of the liver, spleen, kidney and the reproductive organs were increased; however, these generally did not follow a dose-response relationship. Hepatic morphological alterations were not visible after treatment with Compound 5 at either the 20, 50, or 100 mg/kg/day dose, IP for 7 days. After treatment at 100 mg/kg/day, 66% of the sections of the spleen contained perisplenic connective tissue with one focus of red blood cells. At the 20 mg/kg/day dose, one kidney section had a small granulomatous focus in the mesenteric connective tissue. The cortical surface of the kidney contained one small fibrous plaque and one granulomatous focus. The remainder of the kidneys were normal. At the 50 mg/kg/day dose of Compound 5, kidneys showed enlarged pelvic regions lined with transitional epithelial cells with a concomitant cellular loss in the medullar regions. Renal papillae were not seen. Connective tissue at the hilar area had collections of red blood cells (hemorrhagic), which may have been due to post-mortem changes. After 100 mg/kg/day, all kidneys demonstrated changes in the pelvic, medullary, and hilar regions similar to those observed after the 50 mg/kg/day dose.

Serum clinical chemistry values were not elevated after treatment with Compound 5 with the exception of AP, which peaked in mice receiving the 50 mg/kg/day dose. BUN, CP kinase, aldolase, and uric acid levels were reduced significantly at the 20 mg/kg/day dose. Bilirubin levels were reduced in mice receiving the 50 mg/kg/day dose only. Differential white blood cell counts and platelet estimates did not differ from control values for all treatment groups.

Histology of the control animal receiving only the vehicle showed that all the liver sections were normal. The spleen sections showed a few foci of granulomatous inflammation on the capsular surface. One kidney section from a control animal showed a small fibrous plaque on the cortex. These findings were considered incidental.

Enzymatic patterns indicating toxicity in HCT-8 (human small intestine ileum mucosa) cultured cell growth were not altered after incubating these cells with Compounds 1-5 at $10^{-4}$ M (Table VI). Activities of α-glucouronidase, LDH, AP, and trypsin did not differ from control values in both the cell preparation and the medium. No evidence that mucosal villa enzymes leaked from the cells due to drug-induced toxicity was found. Moreover, cytotoxicity was not evident after incubation with the agents at $10^{-4}$ M. $LD_{50}$ values for the five compounds in mice were all greater than 1000 mg/kg, IP. Further testing was difficult because of the limited solubility of the compounds.

**Table VI. The Effects of 2'Deoxyribonucleoside Cyanoboranes on Human HCT-8 Ileum Growth and Enzyme Activities Related to Organ-Specific Toxicity**

| | Percent of Control (Mean ± SD) | | | | | |
|---|---|---|---|---|---|---|
| *N*=6 Compound | Growth (Crystal Violet) | α-Galactosidase | Alkaline Phosphatase | LDH | Trypsin BAEE | Protein Levels |
| 1 | 116± 5 | 108± 7 | 101± 6 | 90± 4 | 100± 6 | 104± 5 |
| 2 | 114± 6 | 97± 6 | 90± 6 | 95± 5 | 99± 5 | 89± 4 |
| 3 | 101± 6 | 95± 7 | 85± 5 | 98± 5 | 100± 5 | 113± 5 |
| 4 | 98± 5 | 113± 6 | 89± 5 | 93± 4 | 102± 6 | 100± 6 |
| 5 | 99± 5 | 95± 4 | 96± 8 | 93± 4 | 101± 6 | 119± 5 |
| Control | 100± 6 | 100± 5 | 100± 6 | 100± 5 | 100± 6 | 100± 6 |

### Discussion

Administration of 2'deoxyribonucleoside cyanoboranes was not toxic to the survival of $CF_1$ mice. The therapeutic dose of these derivations has been established at 8 and 20

mg/kg/day for anti-neoplastic [1,2] and hypolipidemic activity [3]. With all five compounds, very few measured parameters were altered in a way to suggest that this low dose would be toxic under therapeutic conditions.

None of the total body weight and individual organ weight changes at the doses employed for any of the five compounds would be inconsistent with survival of the animals. The clinical chemistry values were not elevated to levels suggesting organ damage induced by the 2'deoxyribonucleoside cyanoboranes. Only treatment with Compound 5 at 100 mg/kg/day suggested any organ-specific toxicity to the kidney, as revealed by histological examination.

The HCT-8 ileum culture assay also indicated that Compounds 1-5 caused no enzymatic leakage from the cells which would suggest organ-specific drug toxicity. The hematocrit (red blood cells) and platelet estimates were normal, suggesting no major effects on the bone marrow hematopoietic system. However, all compounds showed some effects on the differential white blood cell count. In the white blood cell population, more lymphocytes were observed, whereas PMNs were lowered. The total white blood cell population did not appear to be reduced by the agents. Rather, the drug stimulated a shift to more lymphocytes, perhaps in response to the observed increase in inflammation around the spleen mesenteric connective tissue and connective tissue. Perhaps the agents have an immunomodulatory activity that may explain these shifts in white blood cell populations.

The cyanoborane group did not appear to be related to observed histological changes since few changes were observed with Compound 1, which did not contain the cyanoborane moiety. Yet histological examination of tissues after treatment with Compound 2, which contained the cyanoborane moiety and two triisopropylsilyl protecting groups, showed numerous foci of inflammation. However, the parenchymal sections of the organs were generally normal, indicating that the protecting group probably caused no observable tissue damage.

Compounds 3 and 4, cytidine derivatives with triisopropylsilyl protecting groups but no cyanoborane group, demonstrated extensive infection of the spleen and mesentery around the organ. Yet only 5, which contained a cyanoborane group, demonstrated extensive damage to the kidney medullary region and papillae with no damage to liver and spleen. Among the five derivatives tested, Compound 5 was the most active anti-neoplastic agent. It is not unusual for anti-neoplastic agents to cause kidney toxicity, particularly those containing metal complexes, e.g. cis-platinum. Since the boron atom is not considered a heavy metal in the classical sense, it can be inferred that the cyano group caused kidney toxicity. It is interesting to note that the cyanoborane on the guanine 2 did not cause nephrotoxicity, whereas the cytidine derivative 5 was toxic to the kidney. Kidney toxicity was not accompanied by concurrently increased levels of BUN at any dose of Compound 5, which was a peculiar finding. Clinical chemistry values are thought to change before morphological lesions are observable.

**Acknowledgement**
Much appreciation is given to Mr. John Klaric for his assistance in manuscript preparation.

**References**

1. A. Sood, B.F. Spielvogel, B.R. Shaw, L.D. Carlton, B.S. Burnham, E.S. Hall, and I.H. Hall. *Anti-Cancer Res.*, **12** (1992) 335.

2. A. Sood, B.F. Spielvogel, B.R. Shaw, E.S. Hall, L.K. Chi, and I.H. Hall. *der Pharm.*, **47** (1992) 833.
3. I.H. Hall, B.S. Burnham, K.G. Rajendran, S.Y. Chen, A. Sood, and B.F. Spielvogel. *Biomed. & Pharmacotherapy.* In press.
4. I.H. Hall, A.A. Grippo, D.J. Holbrook, J.-J. Chang, L.M.Yang, S.G. Chaney, and K.H. Lee. *Toxicol.* **64** (1990) 203.
5. A. Ness, J. Pastewka, and A.C. Peacock. *Clin. Chem. Acta*, **10** (1964) 229.
6. O.H. Lowry, N.J. Rosebrough, A.L. Farr, and R.J. Randall. *J. Biol. Chem.*, **193** (1951) 263.
7. N. Tietz, *Fundamentals of Clinical Chemistry*, Saunders, Philadelphia, 1976, p. 249.
8. B.A. Brown, *Hematology: Principles and Procedures*, 4th ed., Lea & Febinger, Philadelphia, 1984, p. 29.
9. C.N. Allen, E.S. Harpur, T.J.B. Gray, and B.H. Hirst. *Toxicol. in Vitro*, **5** (1991) 183.
10. H.U. Bergmeyer and E. Bernt. *Methods of Enzymatic Analysis*, **2** (1974) 574.
11. I.H. Hall, C.O. Starnes, K.H. Lee, and T.G. Waddell. *J. Pharm. Sci.*, **69** (1980) 537.
12. P.S. Chen, T.Y. Toribara, and L.T. Warner. *Anal. Chem.* **28** (1956) 1756.
13. W.D. Schleuning and H. Fritz. *Methods in Enzymol.*, **XLV** (1976) 330.
14. H.U. Bergmeyer. *Methods in Enzymol.* (3rd Ed.), **IV** (1984) 228.
15. W. Hasse, A. Schafer, H. Murer, and R. Kinne. *Biochem. J.*, **172** (1978) 57.
16. J.T. Litchfield and F. Wilcoxon. *J. Pharm. Exp. Ther.*, **96** (1949) 99.
17. S.C. Gad and C.S. Weil, *Statistics for toxicologists*, in A.W. Hayes ( Ed.) *Principles and Methods of Toxicology*, 2nd ed., Raven Press, New York, 1982, p. 435.

**Received: May 17, 1993 - Accepted: June 22, 1993**

